# Dual modulation of Ras-Mnk and PI3K-AKT-mTOR pathways: A Novel c-FLIP inhibitory mechanism of 3-AWA mediated translational attenuation through dephosphorylation of eIF4E

**DOI:** 10.1038/srep18800

**Published:** 2016-01-05

**Authors:** Reyaz ur Rasool, Bilal Rah, Hina Amin, Debasis Nayak, Souneek Chakraborty, Abdul Rawoof, Mubashir Javed Mintoo, Khalid Yousuf, Debaraj Mukherjee, Lekha Dinesh Kumar, Dilip Manikaro Mondhe, Anindya Goswami

**Affiliations:** 1 Academy of Scientific & Innovative Research (AcSIR), New Delhi, India; 2 Cancer Pharmacology Division, Indian Institute of Integrative Medicine (CSIR), Canal Road, Jammu Tawi, J&K – 180001, India; 3 Natural Product Chemistry, Indian Institute of Integrative Medicine (CSIR), Canal Road, Jammu Tawi, J&K – 180001, India; 4 Center for Cellular and Molecular Biology, Uppal Road, Hyderabad, AP-50007, India

## Abstract

The eukaryotic translation initiation factor 4E (eIF4E) is considered as a key survival protein involved in cell cycle progression, transformation and apoptosis resistance. Herein, we demonstrate that medicinal plant derivative 3-AWA (from Withaferin A) suppressed the proliferation and metastasis of CaP cells through abrogation of eIF4E activation and expression via c-FLIP dependent mechanism. This translational attenuation prevents the *de novo* synthesis of major players of metastatic cascades *viz.* c-FLIP, c-Myc and cyclin D1. Moreover, the suppression of c-FLIP due to inhibition of translation initiation complex by 3-AWA enhanced FAS trafficking, BID and caspase 8 cleavage. Further ectopically restored c-Myc and GFP-HRas mediated activation of eIF4E was reduced by 3-AWA in transformed NIH3T3 cells. Detailed underlying mechanisms revealed that 3-AWA inhibited Ras-Mnk and PI3-AKT-mTOR, two major pathways through which eIF4E converges upon eIF4F hub. In addition to *in vitro* studies, we confirmed that 3-AWA efficiently suppressed tumor growth and metastasis in different mouse models. Given that 3-AWA inhibits c-FLIP through abrogation of translation initiation by co-targeting mTOR and Mnk-eIF4E, it (3-AWA) can be exploited as a lead pharmacophore for promising anti-cancer therapeutic development.

Translational control plays an important role in cell physiology, including cell growth, proliferation, differentiation and metabolism. The eukaryotic translation initiation factor 4E (eIF4E), an ATP-dependent helicase, is a rate limiting component in the regulation of mRNA translation^1^. The eIF4E interacts with eIF4G, a large scaffold protein for assembly of eIF4F complex at mRNA 7-mGTP cap structure. Formation of eIF4F complex is regulated by a tumor suppressor protein called eIF4E-binding protein 1 (4EBP1) through its reversible association with eIF4E^2^. Mammalian target of rapamycin (mTOR) functionally controls this cap-dependent translation machinery through the phosphorylation of its downstream effectors 4EBPs and S6Ks^3^. Thus, phosphorylation of 4EBP1 by mTOR leads to its association with eIF4E and hence, eIF4F complex formation, whereas inhibition of mTOR results in repression of cap-dependent translation.

Deregulation of eIF4E is a hallmark of many human cancers and its overexpression drives the cells towards malignancy^4^,^5^,^6^. Although the biological significance of eIF4E phosphorylation is not completely understood, a recent study has shown that mice lacking both Mnk1 and Mnk2 developed normally without detectable eIF4E phosphorylation, signifying that Mnk-eIF4E pathway is not an absolute essential for global protein translation but seems extremely important in conditions of stress such as cancer^7^. The eIF4E is considered as one of the pivotal oncogenes and exhibits pro-survival and pro-proliferative activities^8^. Overexpression of eIF4E triggers neoplastic growth largely by initiating the translation of many oncogenic proteins such as c-FLIP, cyclin D1, MMPs, VEGF, c-Myc, HIF1α and ornithine decarboxylase, thereby, facilitating the cancer cells to over-ride the normal growth constrains to invade and maintain the necessary signaling pathways to favour metastatic cascades^4^. A handful of recent literatures demonstrate elevated eIF4E expression in a broad spectrum of human cancer, including prostate, lung, breast, bladder, colon, cervix, neuroblastomas, neck, head, gastrointestinal tract, and Hodgkin’s lymphomas and are often associated with aggressive, poorly differentiated tumors^9^. Importantly, eIF4E knock down by siRNA tremendously impairs the growth of several cancers including those of prostate and breast^10^.

On the other hand, cellular FLICE (FADD-like IL-1β converting enzyme)-inhibitory protein (c-FLIP), a highly ubiquitous protein with a very short half life period, contributes an important role in the CaP metastasis through multi-target effects^11^. Deregulated expression of c-FLIP has been reported in various cancer types and its knock down restores the apoptosis sensitivity of tumor cells^11^. Upregulation of c-FLIP has been evidenced in various tumor types and its (c-FLIP) amplification is closely associated with the development of chemotherapeutic resistance. Nonetheless, knockdown of c-FLIP restores the proapoptotic signaling cascades efficiently (extrinsic as well as intrinsic apoptotic pathways) to enhance chemo-sensitivity^12^.

In our previous study, we have shown that 3-azido Withaferin A (3-AWA), a derivative of Withaferin A, exerts strong anti-proliferative^13^ and anti-invasive effect in prostate and cervical cancer cells by abrogating MMP-2 expression and modulating cellular beta-catenin activity through re-integration of catenin-cadherin complex formation^14^,^15^. In the present study, we examined the effects of 3-AWA mediated controlling of c-FLIP in metastasis/invasion prevention via inhibition of eIF4E phosphorylation involving in the protein translational apparatus.

## Results

### 3-AWA attenuates protein translation initiation

**Figure 1:**
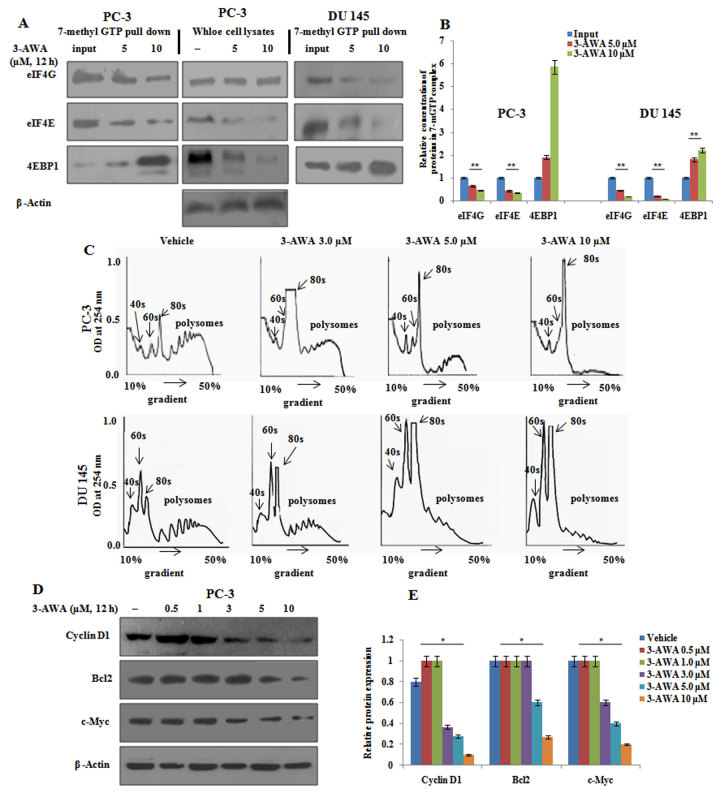
Protein translation initiation attenuation by 3-AWA. (**A**) For cap-binding affinity assay, PC-3 and DU145 cells, were treated with different concentrations of 3-AWA for the set time points. The cell lysates were prepared, incubated with 7m-GTP agarose and bound proteins were determined by western blotting. (**B**) Relative protein concentration determined by densitometry using Image J software. (**C**) Polysomal analysis was performed by treating PC-3 and DU145 cells with different concentrations of 3-AWA. After treatment cell lysates prepared were subjected to 10% to 50% sucrose density gradient ultracentrifugation. Gradients were fractionated and optical density at 254 nm was recorded with an ISCO fractionator. (**D**) PC-3 cells were treated with the indicated doses of 3-AWA for the set time point. Cell lysates prepared were subjected to western blotting for Cyclin D1, Bcl2 and c-Myc proteins probed with respective antibodies. (**E**) Relative protein concentration by densitometry analysis of western blots. Data are mean ± S.D. of three similar experiments. *P < 0.05; **P < 0.01.

We have recently reported 3-AWA suppressed the transcriptional activity of beta catenin along with a distinct modulation of beta catenin sub-cellular distribution in prostate cancer cells^15^. Rationally, as a continuation, here, we examined the role of 3-AWA in translational regulation and further its effect on metastasis prevention in CaP cells. For this, we explored the effect of 3-AWA on translation initiation complex (eIF4F) formation in PC-3, DU145 and BPH-1 cells. The eIF4F complex assembly was analysed by conventional 7m-GTP pull-down assay. In order to do this, PC-3 and DU145 cells treated with 5 μM and 10 μM concentrations of 3-AWA/vehicle following lysis were analysed for eIF4G, eIF4E and 4EBP1 proteins by western blotting. After normalizing eIF4G and 4EBP1 with eIF4E, the binding of eIF4G to eIF4E stably decelerated whereas, 4EBP1 binding to eIF4E increased in a dose dependent manner as compared to vehicle in PC-3 and DU145 cells (Fig. 1A,B). However, negligible disassembly of eIF4F was observed in BPH1 cells (Supplementary Fig. S1A,B). The 7m-GTP pull-down assay was also carried out in PC-3 cells treated with parent withaferin A wherein no dissociation of eIF4F complex was observed in the western blots (supplementary Fig. S2A,B). Data from the pull-down assay was further validated by the polyribosomal analysis. Actively translated mRNA usually contain poly-ribosomes associated with them, whereas mRNA which are translationally inactive are sequestered in messenger ribonucleoprotein particles or are present as monosomes i.e. associated with a single ribosome^16^. In order to perform polysomal assay, PC-3, DU145 and BPH-1 cells were treated with the indicated doses of 3-AWA and/or vehicle for set time points and the respective lysates were fractionated by sucrose gradient to study polysomal and messenger ribonucleoprotein particle fractions. Interestingly, 3-AWA treatment significantly decreased the polysomal RNA fractions and augmented the nonpolysomal or monosomal fractions in PC-3 and DU145 cells (Fig. 1C), while the results were negative in BPH-1 cells (data not shown). We also evaluated the effect of eIF4E down-regulation or translation suppression activity of 3-AWA on the translation of some of the mRNAs, proposed to be selectively regulated by eIF4E. The eIF4E down-regulation concomitantly suppressed cyclin D1, Bcl-2 and c-Myc protein expressions in PC-3 (Fig. 1D,E) clearly demonstrating that 3-AWA suppresses protein translation initiation.

### 3-AWA modulates the function of eIF4E and 4EBP1

**Figure 2:**
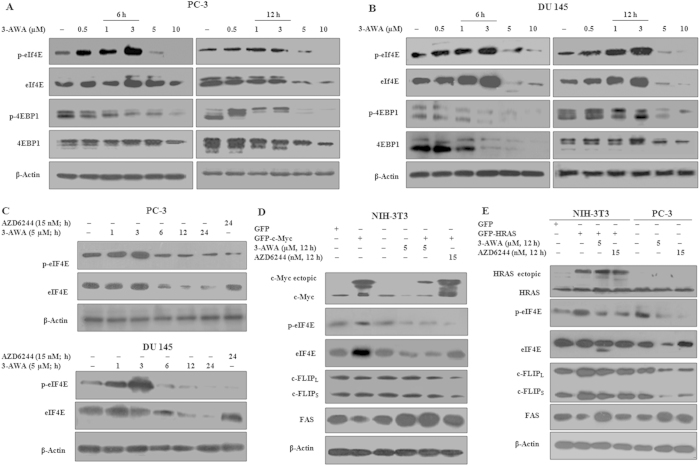
Inhibition of eIF4E and 4EBP1 activity by 3-AWA. (**A**) PC-3 and (**B**) DU145 cells were treated with indicated 3-AWA concentrations for the given time points. Phosphorylation of eIF4E and 4EBP1 was analyzed by western blotting. (**C**) PC-3 and DU145 cells were treated with 3-AWA or AZD6244 for set time points to analyse the phosphorylation and total eIF4E and 4EBP1 status by western blotting. (**D**) The effect of 3-AWA on c-Myc transfected NIH-3T3 cells was checked by transiently transfecting the NIH-3T3 cells with MSCV h c-Myc IRES-GFP or GFP for 48 h, cells were then treated with 3-AWA or AZD6244 or DMSO. The expression of c-Myc, eIF4E, c-FLIP_L/S_ and FAS was probed through western blotting. (**E**) Transient transfection of NIH-3T3 cells with GFP-HRas along with vector GFP for 48 h were performed, followed by treatment with 3-AWA or AZD6244 for indicated time points. PC-3 cell lysates, treated with 3-AWA or AZD6244, were run along with NIH3T3 samples in the same gel as a control. The expression of c-Myc, eIF4E, c-FLIP_L/S_ and FAS proteins was analysed with specific antibodies through western blotting. The blots are representative of three independent results.

To further explore the mechanism of 3-AWA mediated translation suppression, we evaluated the phosphorylation and total expressions of eIF4E (Ser -209), 4EBP1 (Thr-37/46) and eIF4G in 3-AWA treated PC-3 and DU145 cells through western blotting. Our results showed that not only the phosphorylation of eIF4E and 4EBP1 was inhibited but a remarkable down regulation of total eIF4E and 4EBP1 was also obtained as compared to vehicle (Fig. 2A,B). However, there was no change in the expression of eIF4G due to 3-AWA treatment (data not shown). The above effects could be detected as early as 6 h following 3-AWA treatment. The plausible reasons for 4EBP1 downregulation might be either due to its degradation in eIF4E knockdown cells or the vulnerability of its hypophosphorylated form to degradation by KLHL25-CUL3, an E3 ubiquitin ligase complex^1^. A time coarse study unveiled the same pattern of phospho- eIF4E/total- eIF4E in the presence of 3-AWA and AZD6244 (positive control) (Fig. 2C). The phosphorylation and the total expression status of eIF4E were verified with withaferin A, parent molecule of 3-AWA, and no significant changes in eIF4E were found even after 24 h (supplementary Fig. S3A,B).

In contrast to the tight regulation of Myc and Ras in normal cells, their oncogenic activation is frequently seen in various human cancers and is implicated in diverse physiological processes like cellular metabolism, cell proliferation and apoptosis^17^. The eIF4E is one of the important downstream targets of c-Myc and Ras^18^,^19^. Activation of c-Myc is implicated in deregulated expression of the translation initiation factors eIF4E, eIF4A and eIF4G, thereby elevating eIF4F complex formation which in turn has a positive feedback effect on c-Myc expression^20^. Thus eIF4E, which itself is an oncogene, arbitrates the transforming potential of Myc and Ras mutations, thereby causing drug resistance^8^. Rationally, we sought to investigate the effect of 3-AWA on c-Myc and HRas over-expression driven transformation of NIH3T3 cells by analyzing the expression and activation of eIF4E. In order to do this, NIH3T3 cells were transfected with MSCV h c-Myc IRES-GFP and GFP-HRas constructs. Cells with or without transfection were treated with 3-AWA or AZD6244 for the indicated time points. As shown in the Fig. 2D, 3-AWA efficiently negated the c-Myc overexpression mediated upregulation and activation of eIF4E, implying its (3-AWA) target specific inhibitory effects compared to AZD6244 which showed negiligible effect on eIF4E expression. Similarly, it was found that GFP-HRas overexpression increased the overall activity of eIF4E significantly, and this activation of eIF4E was subsequently attenuated by 3-AWA in both H-Ras transfected NIH-3T3 and non transfected PC-3 cells (Fig. 2E). The de-phosphorylation of eIF4E by 3-AWA resulted in a sharp downregulation of c-FLIP along with upregulation of FAS; although the effects were less in NIH-3T3 but were extremely significant in PC-3 cells (Fig. 2E). These results demonstrate that 3-AWA abrogated the phosphorylation of eIF4E and 4EBP1in a time and dose dependent manner.

### 3-AWA inhibits PI3K-dependent and Mnk-mediated eIF4E phosphorylation

**Figure 3:**
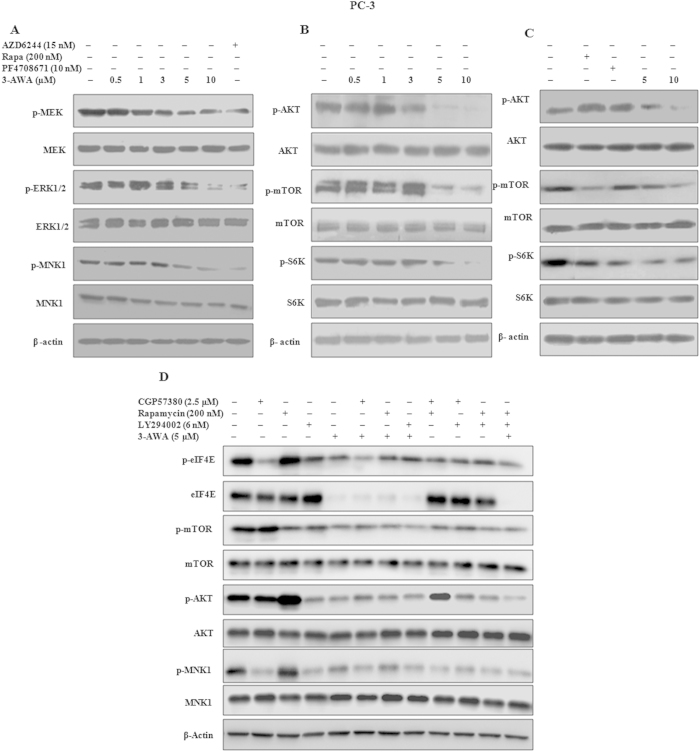
3-AWA co-targets AKT- mTOR and Mnk/eIF4E to attenuate eIF4E phosphorylation. (**A**–**C)** PC-3 cells were treated with indicated concentrations of 3-AWA, PF4708671, Rapamycin and AZD6244 for 12 h. Immunoblot analysis of MEK-ERK1-MNK1 and PI3K-AKT-mTOR-S6K associated proteins was performed in whole cell lysate probed with respective antibodies along with Actin antibody as a loading control. (**D**) PC-3 cells were seeded in 6-well plates. On the next day, cells were treated with the indicated concentrations of 3-AWA, LY294002, CGP57380 or Rapamycin alone and or combination for 12 h. The cells were then subjected to preparation of whole-cell lysates for detection of the indicated proteins using western blotting. The western blots are the representative of three similar experiments.

Translation initiation involves a highly synchronized process. The eIF4E (Ser -209) phosphorylation increases its affinity for mRNA 5′ cap and enhances translation^21^. The eIF4E function is regulated through two main signaling pathways, Ras-MEK1/2-ERK1-MNK1 and PI3K-AKT-mTOR pathways. PI3K-AKT-mTOR pathway activates eIF4E by prevents its interaction with 4EBP1, thereby allowing the translation initiation to occur^22^,^23^,^24^. To envisage the possible mechanism of eIF4E and 4EBP1 (Thr-37/46) dephosphorylation by 3-AWA, we treated PC-3 cells with different concentrations of 3-AWA for particular time points. The total and phosphorylation status of MEK1/2 (Ser-217/221), ERK1 (Thr-202/Tyr-204), MNK1 (Thr-197/202) were analyzed by immunobloting. Interestingly, the results showed that 3-AWA inhibited the phosphorylation of MEK1/2, ERK1, MNK1 at the same concentrations at which the eIF4E (Ser -209) phosphorylation was inhibited (Fig. 3A). We then examined whether 3-AWA also interfered with PI3K-AKT-mTOR signaling pathway. To achieve this objective, 3-AWA treated PC-3 cells were analyzed for phosphorylation status of AKT (Ser-473), mTOR (Ser-2448) and S6K (Thr-389). The results showed that 3-AWA inhibited the phosphorylation of mTOR at the same doses it inhibited 4EBP1 along with expected decrease in phospho S6K level. Intriguingly, irrespective of an increase in phospho- AKT due to mTOR inhibition, as is the case with rapamycin, phosphorylation of AKT attenuated by 3-AWA treatment (Fig. 3B). The plausible explanation for this observation is/may be Par-4 upregulation with 3-AWA treatment^15^. To confirm whether mTOR activity inhibition, accompanied by AKT inhibition was specific to 3-AWA only, PC-3 cells were treated with appropriate doses of rapamycin, an mTOR inhibitor; PF4708671, S6K inhibitor and 3-AWA and or DMSO. The results showed a marked increase in AKT phosphorylation in rapamycin and PF4708671 treated samples when compared to 3-AWA treated samples (Fig. 3C).

Given that the increase in eIF4E phosphorylation might counteract rapamycin’s inhibitory effect on translation initiation^25^, we hypothesized that co-targeting AKT-mTOR and Mnk-eIF4E signaling would result in augmented growth-inhibitory effects in cancer cells. Since we have observed that 3-AWA regulated eIF4E through modulation of Ras-Mnk and PI3K-AKT-mTOR pathways, here, this dual modulation by 3-AWA exhibits an excellent advantage of preventing the increase in eIF4E phosphorylation during mTOR inhibition that might otherwise, counteract mTOR inhibitor’s growth inhibitory effect in cancer cells. To further clarify this dual modulatory effect of 3-AWA, we used different inhibitors of Ras-Mnk and PI3K-AKT-mTOR pathways alone or in combination with 3-AWA (Fig. 3D). Paradoxically, the inhibition of mTOR signaling with rapamycin triggered eIF4E phosphorylation as evidenced by the activation of its activator Mnk1 and importantly 3-AWA abrogated rapamycin’s ability to increase eIF4E and Mnk1 phosphorylation (Fig. 3D). Moreover, mTOR inhibitor Rapamycin also proved its inefficiency to increase eIF4E phosphorylation in presence of CGP57380, Mnk inhibitor, indicating that mTOR inhibitor augmented eIF4E phosphorylation through Mnk-dependent mechanism and 3-AWA followed the same Mnk blocking route to abrogate mTOR’s inhibitory effect on eIF4E hyperactivation (Fig. 3D). Notably, rapamycin also failed to hyperphosphorylate eIF4E in presence of LY294002, PI3K inhibitor, infering that mTOR pathway inhibitors may activate PI3K, leading to an increase in eIF4E phosphorylation. Thus, it is conceivable that 3-AWA inhibits eIF4E phosphorylation through PI3K-dependent and Mnk mediated mechanism. Further, to explore whether 3-AWA is exploiting the aforedescribed route to inhibit cancer cell proliferation and invasion, colony formation and matrigel-invasion assays were performed. The results demonstrate that incubation of CaP cells with 3-AWA or CGP57380 enhanced rapamycin’s ability to inhibit colony formation and invasion compared with the effects of rapamycin alone (supplementary Fig. S3A,B). These results collectively suggest that 3-AWA co-targets AKT- mTOR and Mnk/eIF4E to attenuate eIF4E phosphorylation to restrain growth and invasion of cancer cells

### 3-AWA mediated translational block is associated with c-FLIP downregulation, caspase-8 cleavage and trafficking of FAS via eIF4E

**Figure 4:**
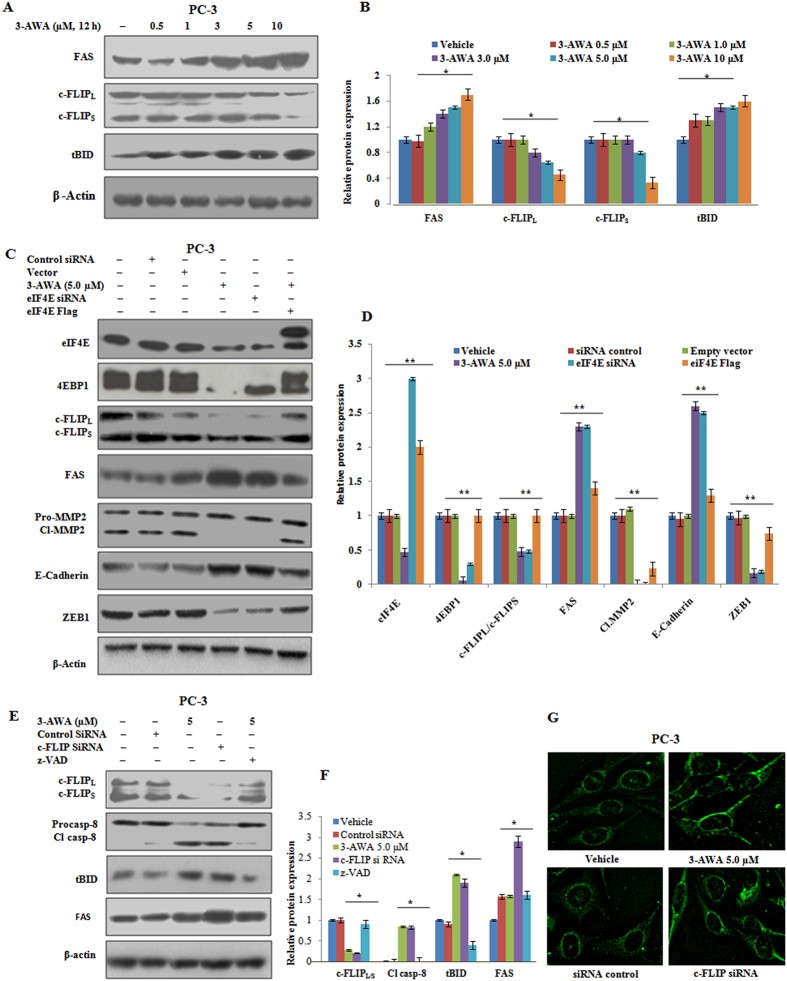
Translational block mediated c-FLIP down-regulation was concomitant with caspase-8, BID cleavage and FAS trafficking. (**A**) PC-3 cells were treated with 3-AWA as shown for the set time periods, cell lysates obtained were subjected to western blotting for FAS, c-FLIP_L/S_ and tBID, probed with respective antibodies. (**B**) The graphs represent the relative protein expression by densitometry analysis of western blots. (**C,D**) PC-3 cells were transiently transfected with either eIF4E Flag DNA/vector or eIF4E siRNA/control siRNA for 48 h. After transfection, cells were treated with 3-AWA for 12 h, cells lysate prepared were subjected to immunoblotting of eIF4E, 4EBP1, c-FLIP_L/S,_ FAS, MMP-2, ZEB1 and E-Cadherin. Relative protein expression by densitometry of western blots. (**E**,**F**) PC-3 cells were transiently transfected with c-FLIP siRNA and control siRNA for 48 h. After transfection, cells were treated with indicated concentrations of 3-AWA in the presence or absence of z-VAD for 12 h. Cells lysates were obtained and subjected to western blotting for c-FLIP_L/S_, caspase-8 and FAS, probed with respective antibodies. (**G**) For immunofluorescence cells were incubated overnight after seeded in 8-well chamber slide. Next day cells were treated with indicated concentrations of 3-AWA in fresh media for 12 h. For the detection of FAS trafficking, the cells were washed with PBS, fixed and incubated with anti-FAS antibody for overnight at 4 °C and successively washed three times following incubation with Alexa Flour 488 conjugated goat anti-rabbit secondary antibody. Accordingly, cells were mounted with ultracruz mounting medium and analyzed with cell imaging station and images were captured at 20x magnification. The data represents the mean value ± SE of three independent experiments. *P < 0.05; **P < 0.01.

It is implicated that knockdown of eIF4E inhibits cancer cell proliferation in both rapamycin sensitive and insensitive cancer cell without activating AKT^24^. Additionally, eIF4E is also essential for the translation of important pro-survival proteins like BCL2, cyclin D1 and c-Myc^26^. We demonstrate here that blocking of protein translation through the inhibition of eIF4E by 3-AWA has a profound effect on c-FLIP. c-FLIP forms an apoptosis inhibitory complex (AIC) by preventing both the formation of death-inducing signaling complex (DISC) and the activation of the caspase cascade^27^. c-FLIP isoforms (c-FLIP_**L,**_c-FLIP_s_ and c-FLIP_**R**_) are also reported to exert a role in the upregulation of many prosurvival and cytoprotective pathways, including ERK1, AKT and NF-kB^28^. To examine the effect of 3-AWA on the expressions of c-FLIP, FAS and tBID, PC-3 cells were treated with different concentrations of the 3-AWA. The western blot results showed decently downregulated expression of c-FLIP isomers and an amplified expression of FAS and tBID in a concentration-dependent manner (Fig. 4A,B). To validate whether the change in expression of c-FLIP was due to 3-AWA mediated translational block (through eIF4E suppression), we performed siRNA mediated eIF4E knockdown and eIF4E over-expression studies. The eIF4E knockdown significantly decreased the c-FLIP, ZEB1 expression and MMP-2 activation along with an enhanced FAS/E-Cadherin expression in presence of 3-AWA; whereas transient eIF4E expression reversed the effect of 3-AWA. Besides the eIF4E dependent down-regulation of c-FLIP and up-regulation of FAS, the decrease in the expression of 4EBP1 was also observed to be dependent on eIF4E in context to 3-AWA treatment (Fig. 4C,D). The above results were also validated by the matrigel invasion assay, wherein a dramatic inhibition in the migration of cancer cells was observed in eIF4E knock-down conditions and eIF4E overexpression consistently restored the invasion event. (supplementary Fig. S4B).

Further, to rule out whether the BID cleavage observed upon 3-AWA treatment was due to c-FLIP-dependent cleavage of caspase-8 or by some other mechanism, PC-3 cells were transfected with c-FLIP siRNA or treated with 3-AWA in presence/absence of z-VAD. The caspase-8 cleavage was robustly enhanced in the c-FLIP siRNA and 3-AWA treated cells as compared to DMSO, control siRNA and 3-AWA plus z-VAD treated cells (Fig. 4E,F). The aim to analyze the expression of truncated BID (t-BID) was to emphasize the activation of both extrinsic and intrinsic apoptotic pathways by 3-AWA following the translational block. Despite the anti-proliferative potency of 3-AWA has already been described in our previous studies^13^,^14^, however, for further support a study of 3-AWA on cell cycle was carried out. The results depicted that 3-AWA arrested the cells in G_1_ stage of the cell cycle (supplementary Fig. S4A). Additionally to cross-check whether the change in expression or activation of eIF4E is independent/dependent of any apoptotic cue, the effect of 3-AWA on eIF4E in presence of z-VAD was examined. It was found that the effect of 3-AWA in context to the activation/expressional changes in eIF4E were consistent (supplementary Fig. S4C), implying that eIF4E regulation due to 3-AWA treatment is independent of cell death and may act as a driver to turn on apoptosis. To further check whether any putative connection existed due to 3-AWA driven reduction in c-FLIP with FAS expression and trafficking, we monitored the expression and trafficking of FAS through immunocytochemistry both in 3-AWA treated and c-FLIP siRNA transfected PC-3 cells. Surprisingly, the results showed a sharp increase in the expression and trafficking of FAS in c-FLIP siRNA transfected and 3-AWA treated cells (Fig. 4G).

### Inhibition of invasion by 3-AWA is c-FLIP dependent

**Figure 5:**
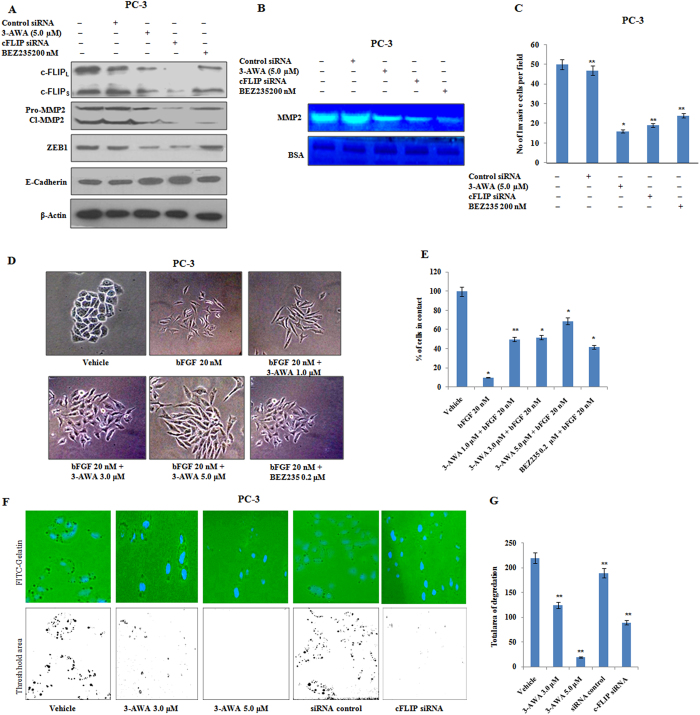
Abrogation of c-FLIP dependent invasiveness of prostate cancer cells by 3-AWA. (**A**) PC-3 cells were transfected with c-FLIP siRNA and control siRNA for 48 h, cells were then treated with indicated concentrations of 3-AWA or BEZ235 for another 12 h. the cell lysates were prepared and subjected to western blotting for the expression of c-FLIP_L/S_, MMP-2, ZEB1 and E-Cadherin proteins with respective antibodies. (**B**) Zymography of MMP-2 along with loading control BSA, after c-FLIP knockdown and/or 3-AWA treatment. (**C**) Cells were transfected with siRNA for c-FLIP or control siRNA, 48 h after transfections the cells were treated with indicated concentrations of 3-AWA for 12 h and matrigel invasion assay was performed. The invaded cells from five random fields in each condition were counted and photographed under an inverted microscope (20 x magnifications). (**D**,**E**) Cells were treated with VEGF alone or in combination with given concentrations of 3-AWA or BEZ235 for 48 h. Cells scattered from individual colony were observed under an inverted microscope and photographed. (**F**,**G**) PC-3 cells were cultured on FITC conjugated gelatin matrix. The threshold areas of degradation were processed and analyzed through Image-J software. Arrows indicate the degradation zone. Data are mean ± S.D. of three similar experiments. *P < 0.05; **P < 0.01.

So far, we have emphasised how 3-AWA influenced translational block and how this translation inhibition rescued the abundance of c-FLIP in PC-3 cells. Although, c-FLIP is known to facilitate tumor cell migration^28^,^29^,^30^, we assumed whether 3-AWA caused any c-FLIP dependent regulation of pro-EMT markers *viz*.; MMPs, ZEB and E-cadherin. As shown in Fig. 5A, 3-AWA attenuated ZEB1 and MMP-2 expression along with an increase in E-Cadherin expression level. Further, to assess the effect of 3-AWA on MMP-2 gelatinase activity, crucial for ECM degradation, we employed gelatin zymography. The results revealed MMP-2 activity was markedly abrogated in 3-AWA treatments and in c-FLIP knock down conditions (Fig. 5B). To further support, *in vitro* matrigel invasion assay showed a considerable decrease in invasion of 3-AWA treated cells as well as in c-FLIP siRNA transfected cells as compared to the control (Fig. 5C). Cell scattering is a characteristic feature of the cells undergoing EMT, and is one of the pivotal initial phenomenon leading to the metastasis^31^. Figure 5D,E demonstrate that 3-AWA inhibited the scattering of the cells, when compared to the vehicle and a positive control (BEZ235) in a dose dependent manner.

Additionally, the ability of prostate cancer cells to degrade the matrix was examined by Fluorescent (FITC)-conjugated-gelatin matrix degradation assay. Figure 5F,G clearly depicted the inhibition of matrix gelatin degradation in aggressive PC-3 cells due to 3-AWA treatment as well as c-FLIP knock down conditions. Threshold areas of degradation obtained from Image-J software, highlighting the degraded area, convincingly supports the inhibition of gelatin matrix degradation. Thus the above results implied that 3-AWA mediated abrogation of invasion and metastasis in prostate cancer was via down regulation of c-FLIP expression.

### *In vivo* anticancer activity against Ehrlich ascites Carcinoma (EAC)

Ehrlich ascites carcinoma is a spontaneous murine mammary adenocarcinoma adapted to ascites form. The experimental results showed that 3-AWA reduced Ehrlich ascites carcinoma by 71.75% at 10 mg/kg intraperitoneal dose in comparison to the tumor growth inhibition of 87.4% in 5-FU (22 mg/kg i.p.) treated group. There was no reduction of weight in the 3-AWA treated group of animals (Table-1). These results demonstrated that 3-AWA as an effective inhibitor of tumor growth in safe and tolerable dose range of 5–10 mg/kg b.w., which was similar in potency with 5-fluorouracil.

### 3-AWA inhibits metastasis and tumor growth *in vivo*

**Figure 6:**
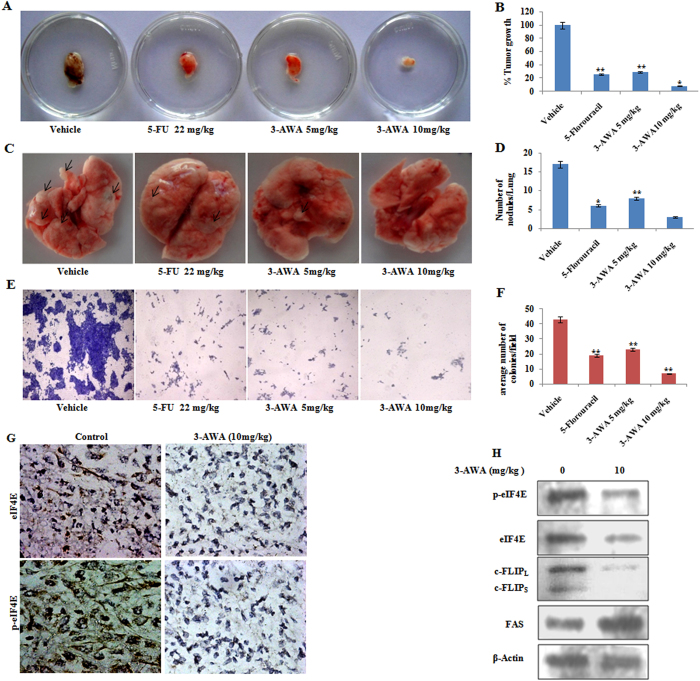
*In-vivo* anti-tumor activities of 3-AWA. (**A**,**B**) 1.5 million 4T1 cells in normal saline were implanted into the mammary pad of each female Balb/c mice. After tumor cell implantation mice were injected i.p. with either normal saline or 3-AWA at 5 mg/kg, 10 mg/kg, and positive control 5-FU, 22 mg/kg body weight every alternate day for two weeks. Animals were sacrificed the tumors were dissected and weighed to check the tumor growth inhibition. (**C**,**D**) Metastatic lung nodules were counted using a dissecting microscope. (**E**,**F**) With the help of 70 μm cell strainer the single cell suspension was obtained from the lungs and serially diluted. Each dilution was resuspended in an appropriate medium containing 6-thioguanine, used for the selection of 4T1 cells. 4T1 cells were allowed to grow and colonies were counted and photographed under fluorescent inverted microscope. (**G**) Tumors excised from controls and treated animals were homogenized and sonicated in lysis buffer. Lysates were boiled with gel loading buffer and loaded on an SDS-polyacrylamide gel and processed for western blotting as shown. (**H**) For immunohistochemical detection of the Phospho-eIF4E and Total eIF4E, paraffin embedded sections from the tumours of treated and untreated mice were incubated with respective primary antibodies and were exposed to 3,3′-diaminobenzidine (DAB) substrate, counterstained with hematoxylin and finally dehydrated and mounted for confocal microscopy. Data are mean ± S.D. of three similar experiments. *P < 0.05; **P < 0.01. One-way ANOVA with SAS 9.2 was carried out to compare the mean of wet tumor weight and number of lung nodules between the six experimental groups.

To validate the anti-metastatic efficacy of 3-AWA, the 4T1 spontaneous/orthotopic mouse mammary carcinoma model was employed. The average body weight of control and 3-AWA-treated mice was monitored throughout the study. In the above model, after the primary tumors developed within the mammary pad of the mice, the mice were treated with 5 mg/kg, 10 mg/kg doses of 3-AWA, negative control (saline) and 5-FU (positive control) on each alternate days for two weeks. After the treatment, the animals were sacrificed; the tumor and lungs were dissected and studied for tumor growth inhibition and formation of metastatic nodules respectively. The primary tumor showed more than 70% and 90% reduction in size, upon treatment with 5 mg/kg and 10 mg/kg groups respectively and this also corresponded to the decrease in the tumor weight at the respective doses (Fig. 6A,B). Thus the mean tumor growth rate at the above doses of 3-AWA were significantly less than negative control, suggesting that at these doses 3-AWA inhibited tumor proliferation, as represented by graphs in Fig. 6B. Supportingly, the number of metastatic lung nodules in 3-AWA-treated mice was considerably reduced in a dose-dependent manner with strong inhibition at 10 mg/kg (Fig. 6C,D). Further, to clarify the anti-metastatic role of 3-AWA, the single cell suspension obtained from the lungs were stained and counted for the respective treatments. It was noticed that the number of cells obtained from the lungs of each treated group corresponded to the number of nodules present, i.e. the number of cells in the flask containing the lung cell suspension, treated with 3-AWA were significantly reduced, with the maximum reduction at 10 mg/kg dose as compared to the control (Fig. 6E,F). To support, we also carried out the western blot and immunohistochemistry analysis of the tumor tissue samples and, as shown in Fig. 6G,H, there was a marked down-regulation in the phospho and total expression of eIF4E resemble our *in vitro* studies.

## Discussion

**Figure 7:**
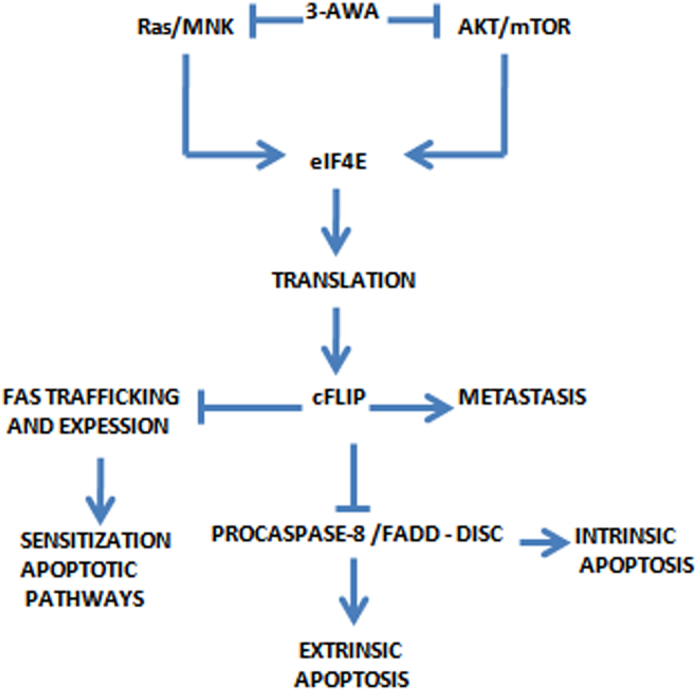
Schematic diagram representing the effect of 3-AWA on translation initiation. 3-AWA treatment results in the blockage of translation initiation by regulating the expression and activation of eIF4E which in turn inhibits the expression of c-FLIP and consequently the invasive behaviour of cancer cells.

Prostate cancer (CaP), a burden to the healthcare system, remains to be the second leading cause of cancer related deaths in the United States and patients with advanced prostate cancer show poor prognosis with severe disease recurrence^32^. Moreover, lack of bonafide molecular targets adaptable to specific therapeutic agents pose a major setback in the treatment of CaP. Therefore, new rational therapeutic approaches need to be established to curb CaP. One of the major potential oncogenic pathway, PI3K-AKT-mTOR pathway, is up-regulated in 40–50% of prostate cancers, often through the loss of PTEN^33^. The other important signaling refers to the Ras-Mnk pathway. Multiple inhibitors of these pathways including bevacizumab, gefitinib, docetaxel, rapamycin and everolimus etc. are either in clinical trials or are presently in the market. Opting for the current conventional approaches is deeply corroborated with many negative distal consequences like hyperactivation of AKT and increased translational rate of oncogenes^33^. Regardless, various cancers, despite their genetic complexity, share a core group of perturbed signaling pathways which converge upon a few regulatory nodes that link these pathways with the basic metabolic machinery of a cell^34^. So it will be at par if we can target a key regulatory node, instead of targeting the genetic alterations individually. One such regulatory node is translation initiation complex eIF4F, which turns multiple signalling pathways, converging upon it through eIF4E, into a self amplifying signalling system and overwhelmingly redirects the translation apparatus of a cell towards the malignancy^22^. 3-azido Withaferin A (3-AWA), a potential derivative of α-β-unsaturated functionality of ring A of withaferin A, has been reported to exert anti-proliferative effect and proven superior over its well-known parent Withaferin A with respect to stall cancer cell invasion. The presence of α-β-unsaturated carbonyl moiety in a plethora of natural products bestow them with an effective chemopreventive and chemoprotective properties, thus renders a high degree of specificity to overcome drug resistance^14^. Although 3-AWA exerts strong anti-proliferative activity in various cancer types, its anti-metastatic effects on aggressive and metastatic CaP cells still needs clarification. In the present study, we investigated the molecular mechanism of 3-AWA’s anti-tumor/anti-metastatic effects in the perspective of translational block as depicted in Fig. 7. We, hereby, report for the first time that 3-AWA attenuated eIF4E induced tumorigenesis by abrogating its expression as well as activation. The eIF4E is an important oncogene for cancer cell survival, growth, angiogenesis and events like epithelial to mesenchymal transition (EMT) that lead to metastasis and is known to be over-expressed in CaP^6^,^35^. Moreover, eIF4E is a rate limiting factor for the assembly of translation initiation complex, eIF4F. Currently, different inhibitors targeting eIF4F are in clinical trials which block the activation of eIF4E by Mnk1 or prevent the association of eIF4E with eIF4G at initiation complex^18^. But no therapeutic agent has been yet reported that could consistently suppress both the expression as well as activation of eIF4E. Regulation of eIF4E involves the two major pro-survival and pro-proliferative pathways, PI3K-Akt-mTOR and Ras-Mnk1 pathways, both of which are perturbed in CaP. Oncogenic Ras mutation overrides the translation initiation control by constantly activating its downstream effector protein Mnk1 to phosphorylate eIF4E through Ras-Mnk1 pathway^25^. Similarly, PI3K-AKT-mTOR pathway also controls the activation of eIF4E oncogene by regulating the phosphorylation of key tumor suppressor protein 4EBP1. 4EBP1, when dephosphorylated interacts strongly with eIF4E to impede its function but is unable to sequester eIF4E when hyperphosphorylated in cancer^36^. Herein, we found that 3-AWA executed its negative regulatory effects on eIF4E in PC-3/DU145 cells, but not in normal prostate BPH-1 cells by consistently blocking Ras-Mnk1and PI3K-AKT-mTOR pathways. Since mTOR inhibitor- induced eIF4E phosphorylation is PI3K and Mnk dependent, one strategy to improve an mTOR inhibitor’s efficacy against cancer is to prevent eIF4E phosphorylation by combining an mTOR inhibitor with a PI3K inhibitor or a Mnk inhibitor. In this study, we have demonstrated that 3-AWA alone exerts similar kind of augmented growth-inhibitory effects in PC-3 cells by blocking AKT-mTOR pathway on one hand and simultaneously debilitates its (3-AWA) mTOR inhibitory effect on eIF4E phosphorylation by impeding Mnk-eIF4E pathway. Notably, the overall expression of eIF4E was affected drastically as was confirmed by down-regulation of eIF4E in c-Myc transfected NIH3T3 cells, suggesting that 3-AWA mediated control of eIF4E is exerted at transcriptional level (data not shown). This depicted the potential of 3-AWA to inhibit eIF4E mediated cancer cell proliferation and metastasis. The importance of this finding can be realized from the fact that over-expression of eIF4E and its hyperactivation by oncogenic alteration in the above mentioned pathways is associated with poor prognosis and relapse-free rate, especially in CaP patients^37^. Furthermore, recently it has been shown that eIF4E is not essential for global protein translation and eIF4E knock down mouse developed normally^38^. Importantly, no hyperactivation of key oncogenes including, AKT and eIF4E was observed *in vitro* with 3-AWA treatment, as has been the case with mTOR inhibitors like rapamycin, where the sudden hyperactivation of AKT and eIF4E in the background negate its anti-tumorigenic effects^38^. As 3-AWA is a strong inducer of pro-apoptotic protein, prostate apoptosis response 4 (Par-4), the plausible reason for the inhibition of AKT might be due to stimulated Par-4 mediated AKT inhibition. Earlier reports have demonstrated profound activation of AKT due to Par-4 ablation in Par-4 KO as well as Par-4/PKCζ DKO cells suggesting Par-4 as a strong selective negative regulator of AKT^15^. Thus, inhibition of eIF4F complex formation assigns 3-AWA a promising chemotherapeutic agent halting eIF4E dependent expression of many pro-metastatic and pro-proliferative proteins including c-FLIP, MMP-2, Cyclin D1, c-Myc and Bcl2.

Additionally, deregulated expression of c-FLIP has been reported in various cancer types and its knock down restores the apoptosis sensitivity of tumor cells^11^. Our finding clearly validates that 3-AWA, through translational block, negatively modulates the expression of c-FLIP and the reliance of this c-FLIP modulation on translation initiation was strongly supported by eIF4E knockdown and over-expression studies. Rationally, the down-regulation of c-FLIP due to the 3-AWA treatment followed by an increase in the FAS trafficking and caspase-8 cleavage paves importance because the FAS expression correlates inversely with the metastatic potential and chemotherapeutic resistance of cancer^11^. However, 3-AWA, by regulating the expression of c-FLIP may switch its anti-apoptotic and pro-proliferative activity to the anti-tumorigenic one^39^. Of note, our *in vivo* results supported the inhibitory activity of 3-AWA on tumor growth and metastasis at as low as 5 mg/kg doses, illustrating moderately less expression of eIF4E/phospho-eIF4E, c-FLIP and elevated FAS expression, which were in accordance with our *in vitro* studies.

Taken together, 3-AWA has a potential to suppress the amplifying oncogenic signals and neoplastic capabilities of a cancer cell by preventing the eIF4F mediated reprogramming of cellular translation apparatus through the blockage of its upstream pathways via eIF4E. Thus, from a clinical cancer treatment point of view, this finding retrospect pharmacological importance of 3-AWA to control deregulated translational machinery.

## Methods

### Cell culture and reagents

The human malignant prostate cancer cell lines PC-3, DU145; normal prostate benign hyperplasia (BPH-1) and normal fibroblast NIH3T3 cells, obtained from Sigma Aldrich (ECACC), were cultured in RPMI 1640 medium (Invitrogen Life Technologies, Carlsberg, CA, USA) supplemented with 10% FBS, 70 mg/L penicillin, 100 mg/L streptomycin, 6 mM HEPES and 2 mM L-glutamine (Sigma-Aldrich, St. Louis, MO, USA) at 37 °C and 5% CO_2_. The major inhibitors used in this study include AZD6244, MEK inhibitor; Rapamycin, mTOR inhibitor; CGP57380, Mnk1/Mnk2 inhibitor; LY294002, PI3K inhibitor; PF4708671, S6K inhibitor were purchased from Selleck chemicals, Houston, USA; BEZ235, AKT inhibitor and z-VAD, caspase inhibitor were purchased from Santa Cruz Biotechnology, Inc., Santa Cruz, CA.

### Western blot analysis

Following the treatments , cell lysates were prepared by lysing the cells in TEGN buffer (10 mM Tris-HCl pH 7.5, 1 mM EDTA, 400 mM NaCl, 0.5% NP-40, and 1 mM DTT) supplemented with protease and phosphatase inhibitors. The cell extractions were centrifuged at 12000 rpm for 10 min at 4 °C. Protein concentration was determined by the standard Bradford method. Equal amounts of proteins were separated in 6–15% SDS-PAGE depending on the molecular weight of the proteins, transferred to PVDF membrane (Millipore) and blocked with 5% non-fat dry milk in PBS/Tween (0.05% Tween-20 in PBS). The antibodies used were anti-eIF4E, antiphospho-eIF4E, anti-4EBP1, antiphospho-4EBP1, anti-ERK11, antiphospho- ERK11, anti-MEK, anti-phospho MEK, anti-mTOR, antiphospho-mTOR, anti- p70S6 kinase α, antiphospho-p70S6 kinase α, anti-cyclin-D1, anti BCL-XL, anti-AKT, antiphospho-AKT and Cleaved Caspase-3 antibody (Cell Signalling Technologies, MA, USA); Caspase-3 (LabMate Asia); anti-eIF4G, anti-Mnk1, antiphospho-Mnk1, anti-eIF2α, antiphospho-eIF2α, anti-FAS, anti-c-FLIP_L/S_, Bcl2 and anti-tBID (Santa Cruz Biotechnology, Inc., Santa Cruz, CA) or with anti-β-actin monoclonal antibody (Sigma-Aldrich, St. Louis, MO, USA) as a control.

For stripping, blots were incubated for 20 min in a buffer containing 62.5 mM Tris/HCl, pH 6.8, 2% SDS and 100 mM β-mercaptoethanol at 37 °C. The blots were washed 3 times for 10 min in PBS. Cell lysates were prepared and western blotting was carried out as described^40^.

### Cap (7m-GTP) pull-down assay

For cap-binding affinity assay, cells were cultured in 60 mm dishes. After treatments with 3-AWA or vehicle, cells were lysed as described above. 15 μl of 7-m GTP beads and 10 μl of CL-4B sepharose beads were mixed, washed with lysis buffer and centrifuged at 7000 rpm for 30 sec to remove the supernatant. Cell lysates (250–400 μg proteins) were added to 7m-GTP-agarose beads, with final volume adjusted to 500 μl, incubated over night at 4 °C. Next day 7m-GTP bound proteins were centrifuged and then washed 4 times using the lysis buffer. The samples were boiled for 5 min in 30 μl of 1X SDS-PAGE sample buffer; the supernatant was collected and loaded into 10–12% SDS PAGE gel.

### Polysome analysis

Polysome profile analysis was carried out as previously described^41^. Briefly, cells were cultured in 60 mm dishes and treated with indicated concentrations of 3-AWA or vehicle for 12 h. Cells were washed with cold PBS containing 100 mg/mL cycloheximide, collected, and lysed in a hypotonic lysis buffer [5 mM Tris-HCl (pH 7.5), 2.5 mM MgCl_2_, 1.5 mM KCl, 100 mg/mL cycloheximide, 2 mmol/L DTT, 0.5% Triton X-100, and 0.5% sodium deoxycholate]. Lysates were added onto 10–50% sucrose density gradients [20 mM HEPES-KOH (pH 7.6), 100 mM KCl, 5 mM MgCl_2_] and centrifuged at 36,000 rpm for 2 h at 4 °C. Gradients were fractionated, and the optical density (OD) at 254 nm was continuously recorded using an ISCO fractionator (Teledyne ISCO).

### Plasmids and transfections

Plasmids expressing eIF4E, c-Myc and HRas or their vectors from addgene (Addgene, Massachusetts, USA) were used. Transfections were carried out with Lipofectamine 2000 or Neon Transfection system (Invitrogen Life Technologies) as per manufacturer’s protocol. After cells were transfected with the required plasmids, the cells with transfections medium were incubated for 4–6 h. After this incubation the transfections medium was replaced with was replaced with complete RPMI 1640 medium and then further incubated for 24 h for expression of the transfected genes.

### siRNA transfection

c-FLIP siRNA was obtained from Santa Cruz Biotechnology, (Santa Cruz) and eIF4E siRNA from Sigma-Aldrich. Briefly, desired number of PC-3 cells were seeded in appropriate dishes or wells and transfected with human specific c-FLIP or eIF4E siRNA. Transfections were performed by using oligofectamine (Invitrogen Life Technologies) according to manufacturer’s instructions.

### Immunocytochemistry

For FAS immunostaining, PC-3 cells were seeded in the Lab-Tek chamber slides (Thermo Scientific, Massachusetts, USA) at a seeding density of 5 × 10^4^ cells/chamber. Following transfection with c-FLIP siRNA for 48 h, cells were treated with different concentrations of 3-AWA or vehicle DMSO for 12 h. After incubation, cells were washed with PBS and fixed in 2.5% paraformaldehyde for 15 min at room temperature, permeabilized with 0.1% Triton-X 100 in PBS for 5 min and successively blocked in 0.5% BSA in PBS for 1 h. For detection of FAS, the cells were incubated with anti rabbit FAS primary antibody (1:200 dilution in blocking buffer) for overnight at 4 °C and next day following incubation with Alexa Fluor 555 (1:500) (Invitrogen Life Technologies) conjugated goat anti-rabbit secondary antibody for 2 h. Accordingly, cells were washed 6 times (5 min each) and mounted with UltraCruz mounting medium (Santacruze) and analyzed with Zeiss LSM-510 metaconfocal microscope under 40x magnification.

### Matrigel invasion assay

Invasion assay was determined using BD Biocoat Tumor Invasion Assay System (BD Bioscience, Bedford, MA) according to the instructions of the manufacturer. Briefly, PC-3 (1.25 × 10^6^) cells were cultured in the presence of 3-AWA or vehicle for 24 h or were transfected with c-FLIP siRNA in serum free media into the upper chambers/inserts of BD Biocoat Tumor Invasion Assay System and the bottom wells were filled with chemoattractant (complete media with 10% FBS). The cells already transfected with c-FLIP siRNA for 48 h were seeded into the chamber. Cells were allowed to migrate at 37 °C. After 24 h, the Matrigel-coated polycarbonate filters were removed, the non-migrating cells were separated from the upper chamber with a cotton swab and the inserts were fixed with methanol and stained with 0.1% crystal violet solution. For each replicate (n = 3), migration of the cells was quantified by counting the stained cells (cells per five fields) under an inverted microscope.

### Cell scatter assay

The cell scattering assay was performed as described previously^42^. Briefly, PC-3 cells were seeded at a density of 1000 cells/well in 6-well plates. After 3-4 days of incubation, small, cohesive, and discrete colonies were formed. Cells were then stimulated with VEGF (20 ng/ml) in the presence of various concentrations of the 3-AWA and/or BEZ235 diluted in growth medium. After 48 h incubation, photographs were taken for individual colonies.

### Invadopodia fluorescent gelatin degradation assay

Coverslips coated with cross-linked-fluorophore FITC-conjugated gelatin matrix were prepared as previously described^43^,^44^. Gelatin-coated coverslips were kept in RPMI containing 10% fetal bovine serum at 37 °C for 2 h prior to cell seeding. To assess the formation of invadopodia and degradation of FITC-gelatin matrix, PC-3 cells treated with 3-AWA or transfected with c-FLIP siRNA, were cultured on coated coverslips. After 24 h of the treatment cells were stained with DAPI mounting and then observed under FLoid Cell Imaging Station (Invitrogen). Threshold area of degradation was determined with ImageJ software.

### Gelatin zymography

The gelatinase activity was assessed following the previously described method from our laboratory^14^. Accordingly, PC-3 cells were transfected and/or treated with indicated conditions. The conditional media obtained after 24 h was employed for zymography. Gelatin zymography of the samples were carried out using 7.5% SDS-polyacrylamide gels containing 0.1% gelatin at 100V for 3 h at 4 °C. Gels were accordingly rinsed with washing buffer (2.0% Triton X-100 in dd water) at room temperature to remove SDS followed by incubation overnight at 37 °C in TCNB buffer (50 mM Tris-HCl, pH 8.0, 10 mM CaCl_2_, 0.02% NaN_3_). Gels were stained with Comassie blue R-250 (Sigma) (0.125% Comassie blue R-250, 50% methanol, 10% acetic acid) for 1–1.5 h and destained with destaining solution (20% methanol, 10% acetic acid, 70% dd water). Gelatinase activity was detected by observing unstained bands on a blue background on Comassie stained gel.

### Flow cytometric cell cycle analysis

Cell cycle analysis was performed as per standard protocol^45^. Briefly, PC-3 cells were seeded in 6 well plates and treated for 12 h. Cells were collected by trypsinization, washed with PBS and incubated in hypotonic solution (0.1% sodium citrate, 25 μg/mL propidium iodide, 0.03% Triton X-100 and 40 μg RNase-A) for 25 min at room temperature in dark. The samples were subsequently analyzed by FACS using BD Diva software

### Immunohistochemistry

For immunohistochemical detection of the phospho-eIF4E and total eIF4E, paraffin embedded sections from the tumors of treated and untreated mice were prepared. Sections were incubated with primary antibodies for phospho-eIF4E and total eIF4E (dilution 1:100), overnight at 4 °C then extensively washed with PBS and further incubated with biotinylated secondary antibody for 1-2 h at room temperature. Sections were exposed to 3,3′-diaminobenzidine (DAB) substrate, counterstained with hematoxylin and finally dehydrated and mounted for confocal microscopy.

### *In vivo* anticancer activity of 3-AWA against Ehrlich ascites carcinoma (EAC)

To evaluate the *in vivo* antitumor activity of 3-AWA, healthy female Swiss albino mice (b.w. 18–23 g) were taken. All the *in vivo* experimental protocols were approved by the Institutional Animal Ethics Committee, CPCSEA, of Indian Institute of Integrative Medicine, Jammu India (IEAC NO. 34/8/14) and the methods were carried out in “accordance” with the approved guidelines. The animals were randomized and divided into three different groups each containing 8 animals. On day first 10 × 10^6^ Sarcoma cells were injected intraperitoneally into the Swiss albino mice (n = 31). The test group was treated with 10 mg/kg i.p. 3-AWA and the positive control group received 22 mg/kg i.p. 5-fluorouracil. The negative control group was administered normal saline (0.2 ml, i.p.). On day 12, animals were sacrificed and ascitic fluid was collected from peritoneal cavity of each mouse for the evaluation of tumour growth. Percent tumor growth inhibition was calculated based on the total number of tumour cells present in the peritoneal cavity as on day 12 of the experiment using the following formula.

(Avg. no of cells in control group – Avg. no of cells in treated group)/(Avg. no of cells in control group) × 100

### *In vivo* anti-metastasis and anti-tumor efficacy studies

Healthy, female Balb/c mice (5–7 weeks old) were taken for the study. For subcutaneous implantations, the mice were randomized into four groups of 10 mice per group and 1.5 million 4T1 cells in serum free RPMI media were implanted into the mammary pad of each mouse. A week after tumor cell implantation, mice were injected intraperitoneally with either vehicle (saline) or 3-AWA at 5 mg/kg, 10 mg/kg, and positive control 5-FU 22 mg/kg body weight in every alternate day for two weeks. Tumor sizes were recorded after every 2 or 3 days after cell injection and body weight was recorded once per week. Mice were sacrificed after 15 days of the treatment initiation and metastatic lung nodules were counted using a dissecting microscope. Then, with the help of 70 μm cell strainer (BD, USA) the single cell suspension obtained from the lungs were serially diluted for 3-4 times and each dilution was resuspended in a selection medium containing 6-thioguanine, used for the selection of 4T1 cells. Tumors were dissected and growth was measured. Tumors excised from control and treated animals were homogenized and sonicated in lysis buffer [50 mM Tris-HCl, pH 7.5, 150 mM NaCl, 1% NP-40, 1 mM EDTA, 0.25% sodium deoxycholate, 1 mM NaF, 1 mM sodium orthovanadate and complete protease inhibitor (Roche, USA)]. Lysates were prepared and processed for western blotting. One-way ANOVA with SAS 9.2 was carried out to compare the mean of wet tumor weight and number of lung nodules between the four experimental groups.

### Statistical analysis

Data were expressed as the mean ± standard error of three independent experiments and analysed by the student’s t test. A two sided value of p < 0.05 was considered significant in all cases.

## Additional Information

**How to cite this article**: ur Rasool, R. *et al.* Dual modulation of Ras-Mnk and PI3K-AKT-mTOR pathways: A Novel c-FLIP inhibitory mechanism of 3-AWA mediated translational attenuation through dephosphorylation of eIF4E. *Sci. Rep.* **6**, 18800; doi: 10.1038/srep18800 (2016).

## Supplementary Material

Supplementary Information

## Figures and Tables

**Table 1:** *In vivo* anti cancer activity of 3-AWA.

Treatment groups	Avg. body weights (g) of animals on days			12^th^ day						
	1^st^	5^th^	9^th^	Av. body weights (g)	Avg. Volume of ascitic fluid (ml)	Av. weight of ascitic fluid (g)	Av. no. of tumor cells (10^7^)	% tumor cell growth	% tumor growth inhibition	Mortality
3-AWA (10 mg/kg i.p.)	21.52	22.32	21.45	21.76	3.11 ± .88	2.93 ± .87	37.8 ± 2.29*	29.5	70.5	0/8
5-FU (22 mg/kg i.p.)	22.85	21.22	20.34	21.47	1.43 ± 0.21	1.13 ± 0.15	31.5 ± 0.78**	24.6	87.4	0/8
Normal saline (0.2 ml i.p.)	24.22	22.16	23.13	23.17	9.76 ± 0.30	9.73 ± 0.69	128.42 ± 9.87	100	—	0/8

Values are mean ± S.E. (n = 7, 10 for control). *P < 0.05, **P < 0.01 versus normal saline control. Comparisons were made between the control and treated groups using the Student’s t-test.

## References

[b1] Yanagiya A. *et al.* Translational homeostasis via the mRNA cap-binding protein, eIF4E. Molecular cell 46, 847–858 (2012). 22578813 10.1016/j.molcel.2012.04.004 PMC4085128

[b2] Richter J. D. & Sonenberg N. Regulation of cap-dependent translation by eIF4E inhibitory proteins. Nature 433, 477–480 (2005). 15690031 10.1038/nature03205

[b3] Hsieh A. C. *et al.* Genetic dissection of the oncogenic mTOR pathway reveals druggable addiction to translational control via 4EBP-eIF4E. Cancer cell 17, 249–261 (2010). 20227039 10.1016/j.ccr.2010.01.021 PMC2901095

[b4] Mamane Y. *et al.* eIF4E-from translation to transformation. Oncogene 23, 3172–3179 (2004). 15094766 10.1038/sj.onc.1207549

[b5] Raught B. & Gingras A.-C. eIF4E activity is regulated at multiple levels. The international journal of biochemistry & cell biology 31, 43–57 (1999). 10216943 10.1016/s1357-2725(98)00131-9

[b6] Robichaud N. *et al.* Phosphorylation of eIF4E promotes EMT and metastasis via translational control of SNAIL and MMP-3. Oncogene 34, 2032–2042 (2014). 24909168 10.1038/onc.2014.146 PMC4978545

[b7] Ueda T., Watanabe-Fukunaga R., Fukuyama H., Nagata S. & Fukunaga R. Mnk2 and Mnk1 are essential for constitutive and inducible phosphorylation of eukaryotic initiation factor 4E but not for cell growth or development. Molecular and cellular biology 24, 6539–6549 (2004). 15254222 10.1128/MCB.24.15.6539-6549.2004 PMC444855

[b8] Lazaris-Karatzas A., Montine K. S. & Sonenberg N. Malignant transformation by a eukaryotic initiation factor subunit that binds to mRNA 5′cap. Nature 345, 544–547 (1990). 2348862 10.1038/345544a0

[b9] De Benedetti A. & Graff J. R. eIF-4E expression and its role in malignancies and metastases. Oncogene 23, 3189–3199 (2004). 15094768 10.1038/sj.onc.1207545

[b10] Soni A. *et al.* eIF4E knockdown decreases breast cancer cell growth without activating Akt signaling. Molecular Cancer Therapeutics 7, 1782–1788 (2008). 18644990 10.1158/1535-7163.MCT-07-2357 PMC2559956

[b11] Safa A. R. c-FLIP, a master anti-apoptotic regulator. Exp Oncol 34, 176–184 (2012). 23070002 PMC4817998

[b12] Safa A. R. Roles of c-FLIP in Apoptosis, Necroptosis, and Autophagy. Journal of carcinogenesis & mutagenesis 6, 003 (2013). 25379355 10.4172/2157-2518.S6-003 PMC4219646

[b13] Rah B. *et al.* PAWR-mediated suppression of BCL2 promotes switching of 3-azido withaferin A (3-AWA)-induced autophagy to apoptosis in prostate cancer cells. Autophagy 11, 314–331 (2015). 25803782 10.1080/15548627.2015.1017182 PMC4502794

[b14] Rah B. *et al.* A novel MMP-2 inhibitor 3-azidowithaferin A (3-azidoWA) abrogates cancer cell invasion and angiogenesis by modulating extracellular Par-4. PloS one 7, e44039 (2012). 22962598 10.1371/journal.pone.0044039 PMC3433490

[b15] Amin H. *et al.* Par-4 dependent modulation of cellular β-catenin by medicinal plant natural product derivative 3-azido Withaferin A. Molecular Carcinogenesis (2015), 10.1002/mc.22328. 25969134

[b16] Kelen K. V. D., Beyaert R., Inzé D. & Veylder L. D. Translational control of eukaryotic gene expression. Critical reviews in biochemistry and molecular biology 44, 143–168 (2009). 19604130 10.1080/10409230902882090

[b17] Waskiewicz A. J. *et al.* Phosphorylation of the cap-binding protein eukaryotic translation initiation factor 4E by protein kinase Mnk1 *in vivo*. Molecular and cellular biology 19, 1871–1880 (1999). 10022874 10.1128/mcb.19.3.1871 PMC83980

[b18] Bitterman P. B. & Polunovsky V. A. Attacking a nexus of the oncogenic circuitry by reversing aberrant eIF4F-mediated translation. Molecular Cancer Therapeutics 11, 1051–1061 (2012). 22572598 10.1158/1535-7163.MCT-11-0530 PMC3349966

[b19] Ruggero D. The role of Myc-induced protein synthesis in cancer. Cancer research 69, 8839–8843 (2009). 19934336 10.1158/0008-5472.CAN-09-1970 PMC2880919

[b20] Lin C.-J., Cencic R., Mills J. R., Robert F. & Pelletier J. c-Myc and eIF4F are components of a feedforward loop that links transcription and translation. Cancer research 68, 5326–5334 (2008). 18593934 10.1158/0008-5472.CAN-07-5876

[b21] Mochizuki K., Oguro A., Ohtsu T., Sonenberg N. & Nakamura Y. High affinity RNA for mammalian initiation factor 4E interferes with mRNA-cap binding and inhibits translation. Rna 11, 77–89 (2005). 15611299 10.1261/rna.7108205 PMC1370693

[b22] Meric F. & Hunt K. K. Translation Initiation in Cancer: A Novel Target for Therapy 1 FM is supported by The University of Texas MD Anderson Cancer Center Physician-Scientist Program and by NIH Grant 1KO8-CA 91895-01. KKH is supported by Department of Defense Award DAMD-17-97-1-7162. 1. Molecular Cancer Therapeutics 1, 971–979 (2002). 12481419

[b23] McCubrey J. A. *et al.* Mutations and deregulation of Ras/Raf/MEK/ERK and PI3K/PTEN/Akt/mTOR cascades which alter therapy response. Oncotarget 3, 954 (2012). 23006971 10.18632/oncotarget.652 PMC3660063

[b24] Gingras A.-C. *et al.* Regulation of 4E-BP1 phosphorylation: a novel two-step mechanism. Genes & development 13, 1422–1437 (1999). 10364159 10.1101/gad.13.11.1422 PMC316780

[b25] Sun S.-Y. *et al.* Activation of Akt and eIF4E survival pathways by rapamycin-mediated mammalian target of rapamycin inhibition. Cancer research 65, 7052–7058 (2005). 16103051 10.1158/0008-5472.CAN-05-0917

[b26] Mamane Y., Petroulakis E., LeBacquer O. & Sonenberg N. mTOR, translation initiation and cancer. Oncogene 25, 6416–6422 (2006). 17041626 10.1038/sj.onc.1209888

[b27] Quintavalle C. *et al.* c-FLIPL enhances anti-apoptotic Akt functions by modulation of Gsk3β activity. Cell Death & Differentiation 17, 1908–1916 (2012). 20508645 10.1038/cdd.2010.65

[b28] Kataoka T. *et al.* The caspase-8 inhibitor FLIP promotes activation of NF-kB and Erk signaling pathways. Current Biology 10, 640–648 (2000). 10837247 10.1016/s0960-9822(00)00512-1

[b29] Maedler K. *et al.* FLIP switches Fas-mediated glucose signaling in human pancreatic β cells from apoptosis to cell replication. Proceedings of the National Academy of Sciences 99, 8236–8241 (2002). 10.1073/pnas.122686299 PMC123051 12060768

[b30] Fang L.-W., Tai T.-S., Yu W.-N., Liao F. & Lai M.-Z. Phosphatidylinositide 3-kinase priming couples c-FLIP to T cell activation. Journal of Biological Chemistry 279, 13–18 (2004). 14578361 10.1074/jbc.M303860200

[b31] Fram S. T., Wells C. M. & Jones G. E. HGF-induced DU145 cell scatter assay, In Cell Migration 31–40 (Springer, 2011). 10.1007/978-1-61779-207-6_3 21748667

[b32] Yu Y. P. *et al.* Identification of a novel gene with increasing rate of suppression in high grade prostate cancers. The American journal of pathology 158, 19–24 (2001). 11141474 10.1016/S0002-9440(10)63939-9 PMC1850281

[b33] Galazi M., Rodriguez-Vida A., Ng T., Mason M. & Chowdhury S. Precision medicine for prostate cancer. Expert review of anticancer therapy 14, 1305–1315 (2014). 25354871 10.1586/14737140.2014.972948

[b34] Bitterman P. B. & Polunovsky V. A. Translational control of cell fate: from integration of environmental signals to breaching anticancer defense. Cell Cycle 11, 1097–1107 (2012). 22356766 10.4161/cc.11.6.19610

[b35] Grzmil M. & Hemmings B. A. Translation regulation as a therapeutic target in cancer. Cancer research 72, 3891–3900 (2012). 22850420 10.1158/0008-5472.CAN-12-0026

[b36] Faller W. J. *et al.* mTORC1-mediated translational elongation limits intestinal tumour initiation and growth. Nature 517, 497–500 (2015). 25383520 10.1038/nature13896 PMC4304784

[b37] Bianchini A. *et al.* Phosphorylation of eIF4E by MNKs supports protein synthesis, cell cycle progression and proliferation in prostate cancer cells. Carcinogenesis 29, 2279–2288 (2008). 18809972 10.1093/carcin/bgn221

[b38] Schwarzer A. *et al.* Hyperactivation of mTORC1 and mTORC2 by multiple oncogenic events causes addiction to eIF4E-dependent mRNA translation in T-cell leukemia. Oncogene 34, 3593–3604 (2014). 25241901 10.1038/onc.2014.290

[b39] Zhang X. *et al.* Persistent c-FLIP (L) expression is necessary and sufficient to maintain resistance to tumor necrosis factor-related apoptosis-inducing ligand-mediated apoptosis in prostate cancer. Cancer research 64, 7086–7091 (2004). 15466204 10.1158/0008-5472.CAN-04-1498

[b40] Alain T. *et al.* Vesicular stomatitis virus oncolysis is potentiated by impairing mTORC1-dependent type I IFN production. Proceedings of the National Academy of Sciences 107, 1576–1581 (2010). 10.1073/pnas.0912344107 PMC2824402 20080710

[b41] Dowling R. J. O., Zakikhani M., Fantus I. G., Pollak M. & Sonenberg N. Metformin inhibits mammalian target of rapamycin-dependent translation initiation in breast cancer cells. Cancer research 67, 10804–10812 (2007). 18006825 10.1158/0008-5472.CAN-07-2310

[b42] Ghosh S., Basu M. & Roy S. S. ETS-1 protein regulates vascular endothelial growth factor-induced matrix metalloproteinase-9 and matrix metalloproteinase-13 expression in human ovarian carcinoma cell line SKOV-3. Journal of Biological Chemistry 287, 15001–15015 (2012). 22270366 10.1074/jbc.M111.284034 PMC3340257

[b43] Martin K. H. *et al.* Quantitative measurement of invadopodia-mediated extracellular matrix proteolysis in single and multicellular contexts. Journal of visualized experiments: JoVE 66, e4119 (2012). 22952016 10.3791/4119 PMC3606055

[b44] Hoover H., Muralidharan Chari V., Tague S. & D’Souza Schorey C. Investigating the Role of ADP Ribosylation Factor 6 in Tumor Cell Invasion and Extracellular Signal Regulated Kinase Activation. Methods in enzymology 404, 134–147 (2005). 16413265 10.1016/S0076-6879(05)04014-0

[b45] Pozarowski P. & Darzynkiewicz Z. Analysis of cell cycle by flow cytometry, In Checkpoint Controls and Cancer 301–311 (Springer, 2004). 10.1385/1-59259-811-0:301 15220539

