# Corrigendum: Dual modulation of Ras-Mnk and PI3K-AKT-mTOR pathways: A Novel c-FLIP inhibitory mechanism of 3-AWA mediated translational attenuation through dephosphorylation of eIF4E

**DOI:** 10.1038/srep21054

**Published:** 2016-02-17

**Authors:** Reyaz ur Rasool, Bilal Rah, Hina Amin, Debasis Nayak, Souneek Chakraborty, Abdul Rawoof, Mubashir Javed Mintoo, Khalid Yousuf, Debaraj Mukherjee, Lekha Dinesh Kumar, Dilip Manikaro Mondhe, Anindya Goswami

Scientific Reports 6: Article number: 18800; 10.1038/srep18800 published online: 01052016; updated: 02172016.

This Article contains typographical errors.

In the Results section under subheading ‘*In vivo* anticancer activity against Ehrlich ascites Carcinoma (EAC)’

“The experimental results showed that 3-AWA reduced Ehrlich ascites carcinoma by 71.75% at 10 mg/kg intraperitoneal dose in comparison to the tumor growth inhibition of 87.4% in 5-FU (22 mg/kg i.p.) treated group.”

should read:

“The experimental results showed that 3-AWA reduced Ehrlich ascites carcinoma by 70.5% at 10 mg/kg intraperitoneal dose in comparison to the tumor growth inhibition of 87.4% in 5-FU (22 mg/kg i.p.) treated group.”

In the legend of Table 1

“Values are mean ± S.E. (n = 7, 10 for control).”

should read:

“Values are mean ± S.E. (n = 8).”

