# Supplementary material for: Dual modulation of Ras-Mnk and PI3K-AKT-mTOR pathways: A Novel c-FLIP inhibitory mechanism of 3-AWA mediated translational attenuation through dephosphorylation of eIF4E
Source: Sci Rep. 2016 Jan 5;6:18800. doi: 10.1038/srep18800 (PMC4700468; doi:10.1038/srep18800)
Supplement: Supplementary Information [file srep18800-s1.doc]

**Dual modulation of Ras-Mnk and PI3K-AKT-mTOR pathways: Novel c-FLIP inhibitory mechanism of 3-AWA** **mediated translational attenuation through dephosphorylation of eIF4E**

Reyaz ur Rasool^a,b^, Bilal Rah^a,b^, Hina Amin^b^, Debasis Nayak^a,b^, Souneek Chakraborty^a,b^, Abdul Rawoof^d^, Mubashir Minto^a,b^, Khalid Yousuf ^c^, Debaraj Mukherjee^c^, Lekha Dinesh Kumar^d^, Dilip Manikaro Mondhe ^a,b^*, Anindya Goswami^a,b^*

^a^Academy of Scientific & Innovative Research (AcSIR), New Delhi, India

^b^Cancer Pharmacology Division, Indian Institute of Integrative Medicine (CSIR), Canal Road, Jammu Tawi, J&K – 180001, India.

^c^Natural Product Chemistry, Indian Institute of Integrative Medicine (CSIR), Canal Road, Jammu Tawi, J&K – 180001, India.

^d^Center for Cellular and Molecular Biology, Uppal Road, Hyderabad, AP-50007, India.

***Corresponding Authors**

Dr. Anindya Goswami, Cancer Pharmacology Division, Indian Institute of Integrative Medicine (CSIR), Jammu Tawi-180001, India.Telephone: 0191-2569111, Fax: 0191-2569333, Email: [agoswami@iiim.ac.in](mailto:agoswami@iiim.ac.in)

Dr. Dilip Manikaro Mondhe, Cancer Pharmacology Division, Indian Institute of Integrative Medicine (CSIR), Jammu Tawi - 180001, India. Telephone: 09419131776, Email: [dmmondhe@iiim.ac.in](mailto:dmmondhe@iiim.ac.in)

**
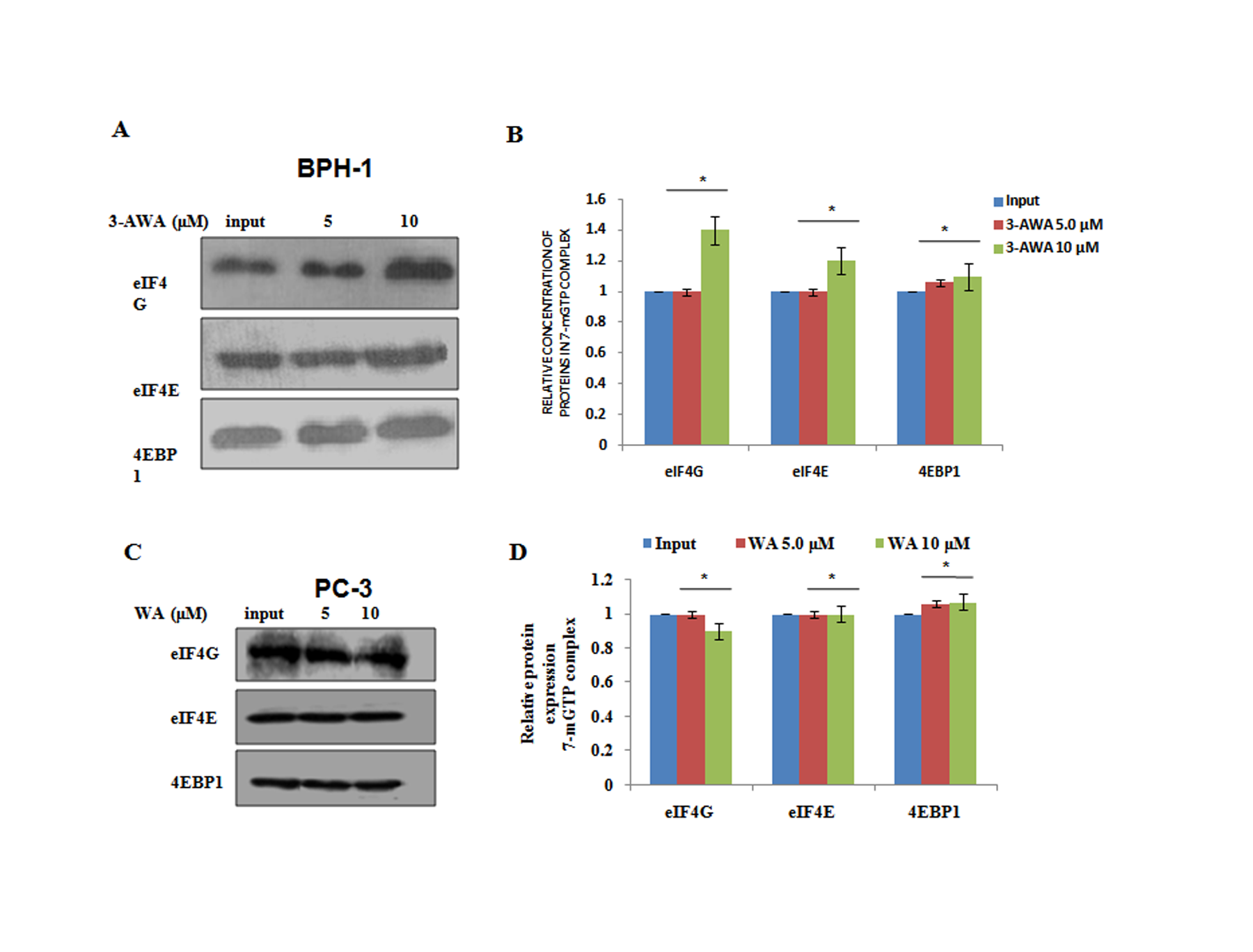
**

**Supplementary Figure 1. Effect of 3-azido withaferin A (3-AWA) and its parent compound Withaferin A (WA) on the translation initiation complex assembly in BPH-1 and PC-3 cells.** (**A and B**) After treatment with different concentrations of 3-AWA for indicating time point, lysates were prepared from BPH-1 cells and 7m-GTPagarose pull down assay was performed as described in material methods. (**C and D**) After treatment with different concentrations of withaferin A for the set time points, lysates were prepared from PC-3 cells and incubated with 7m-GTPagarose. 7m-GTP bound proteins were denatured in SDS loading buffer and detected by western blotting. Relative protein expression by densitometry analysis of western blots. **P < 0.05.*


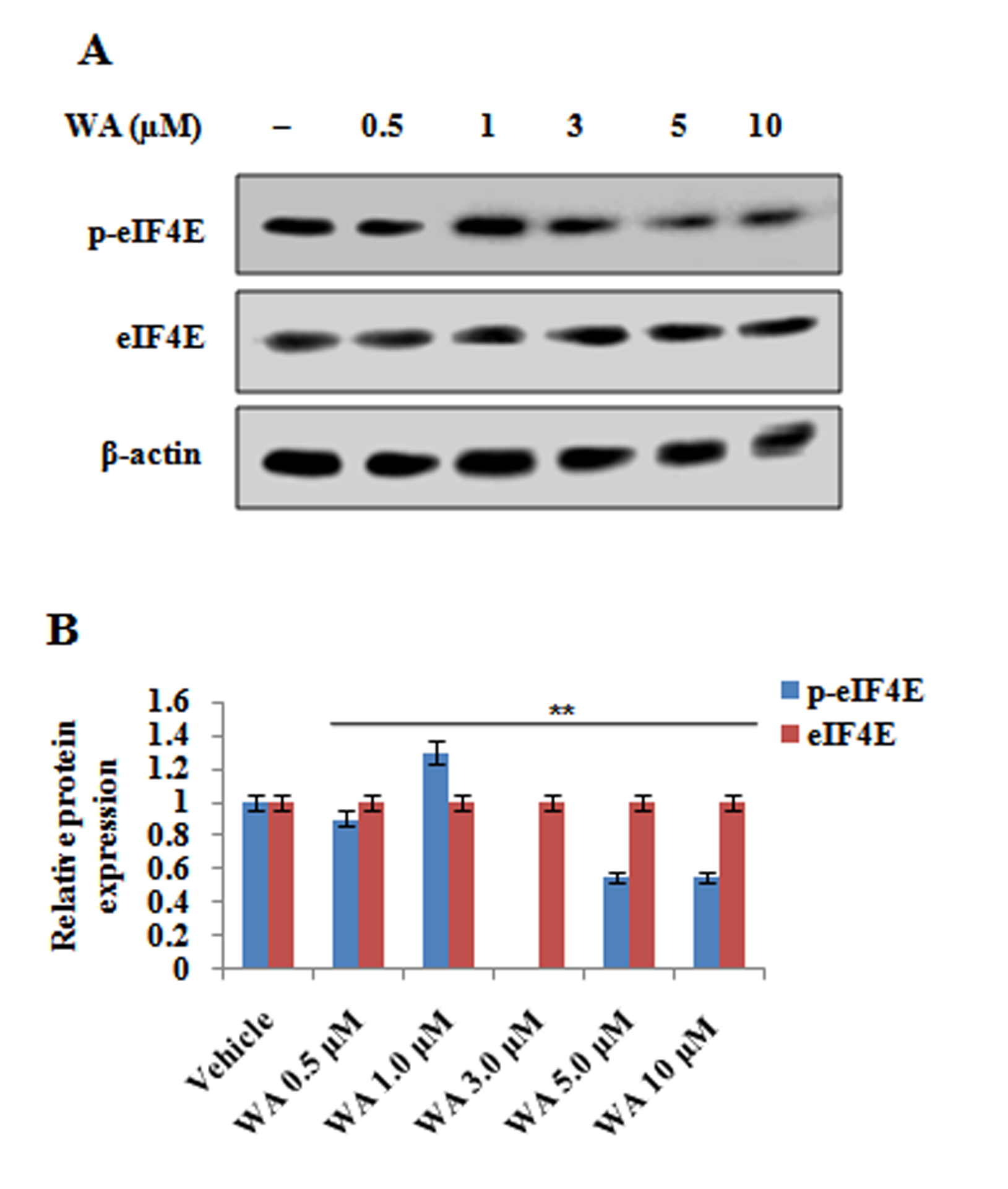


**Supplementary Figure 2. Withaferin A (WA) has no or insignificant effect on eIF4E expression and activation.** (**A**) PC-3 cells were treated with WA as indicated for 24 h. p-eIF4E and eIF4E expression was detected through western blotting. (**B**) Densitometry analysis, representation of western blots. ***P < 0.01.*


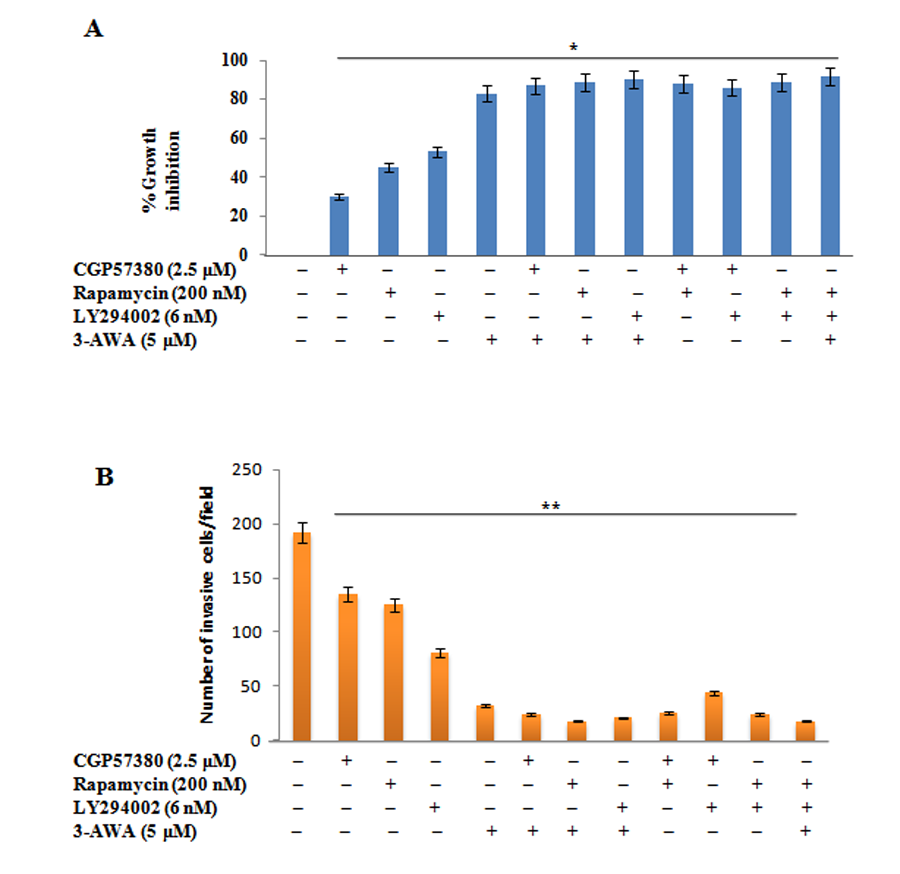


**Supplementary Figure 3. Inhibition of Mnk-dependent eIF4E phosphorylation by 3-AWA thwart metastasis and proliferation in human prostate cancer cells.** **(A)** PC-3 cells at a density of 1000 cells per well were seeded in 35 mm plates. Next day, the cells were treated with the indicated concentrations of 3-AWA, LY294002, CGP57380, Rapamycin alone and/or in combination. The same treatments were repeated every 3 days. After 10 days, the plates were stained for the formation of cell colonies with crystal violet. The colonies in each well were counted. **(B)** PC-3 cells were treated as shown for 24 h and matrigel invasion assay was performed. The invaded cells from five random fields in each condition were counted and photographed under an inverted microscope (20 x magnifications). The data represents the mean value ± SE of three independent experiments. **P < 0.05; **P < 0.01.*

**
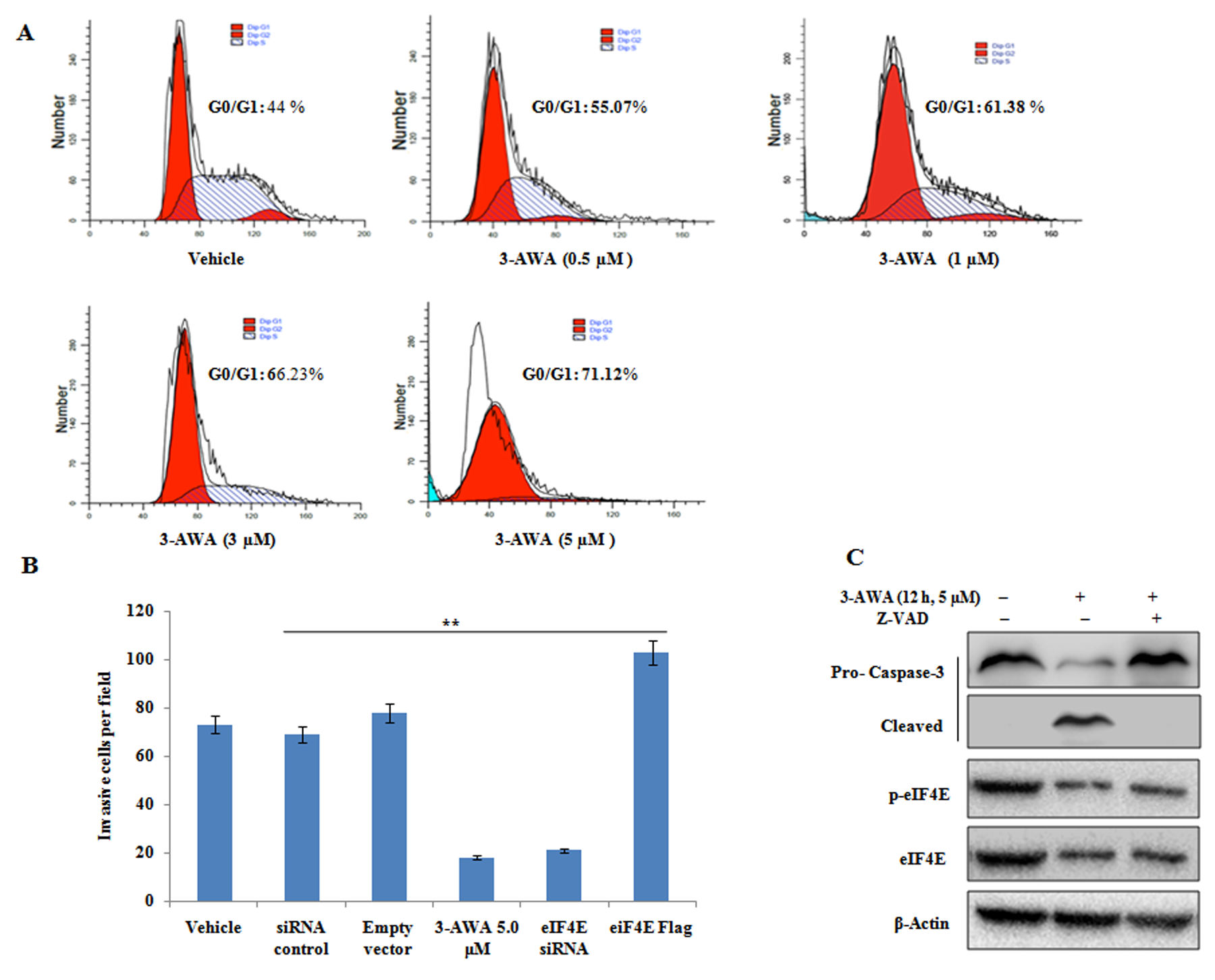
**

**Supplementary Figure 4. 3-AWA arrests PC-3 cells in G1 phase and induces apoptosis. (A)** 3-AWA along with vehicle DMSO treatment was given to the PC-3 cells for 12 h and were analyzed by FACS, (as indicated); graphical representation show the percentage of cells in G0/G phase. **(B)** PC-3 cells were treated as shown for 24 h and matrigel invasion assay was performed. The invaded cells from five random fields in each condition were counted and photographed under an inverted microscope (20 x magnifications). **(C)** Autoradiographs show the cleavage and expression of caspase-3 and eIF4E (phospho and total) in PC-3 cells when treated with 3-AWA and/or z-VAD. The data represents the mean value ± SE of three independent experiments. **P < 0.05; **P < 0.01.*
